# Ethical implications of AI and robotics in healthcare: A review

**DOI:** 10.1097/MD.0000000000036671

**Published:** 2023-12-15

**Authors:** Chukwuka Elendu, Dependable C. Amaechi, Tochi C. Elendu, Klein A. Jingwa, Osinachi K. Okoye, Minichimso John Okah, John A. Ladele, Abdirahman H. Farah, Hameed A. Alimi

**Affiliations:** a University of California, Santa Cruz, CA; b Igbinedion University, Okada, Nigeria; c Imo State University, Owerri, Nigeria; d Kazan State Medical University, Kazan, Russia; e Chukwuemeka Odumegwu Ojukwu University Teaching Hospital, Awka, Nigeria; f Babcock University, Ilishan-Remo, Nigeria; g Luhansk State Medical University, Luhansk, Ukraine.

**Keywords:** algorithmic bias, artificial intelligence (AI), data security, ethical considerations, healthcare, privacy, robotics

## Abstract

Integrating Artificial Intelligence (AI) and robotics in healthcare heralds a new era of medical innovation, promising enhanced diagnostics, streamlined processes, and improved patient care. However, this technological revolution is accompanied by intricate ethical implications that demand meticulous consideration. This article navigates the complex ethical terrain surrounding AI and robotics in healthcare, delving into specific dimensions and providing strategies and best practices for ethical navigation. Privacy and data security are paramount concerns, necessitating robust encryption and anonymization techniques to safeguard patient data. Responsible data handling practices, including decentralized data sharing, are critical to preserve patient privacy. Algorithmic bias poses a significant challenge, demanding diverse datasets and ongoing monitoring to ensure fairness. Transparency and explainability in AI decision-making processes enhance trust and accountability. Clear responsibility frameworks are essential to address the accountability of manufacturers, healthcare institutions, and professionals. Ethical guidelines, regularly updated and accessible to all stakeholders, guide decision-making in this dynamic landscape. Moreover, the societal implications of AI and robotics extend to accessibility, equity, and societal trust. Strategies to bridge the digital divide and ensure equitable access must be prioritized. Global collaboration is pivotal in developing adaptable regulations and addressing legal challenges like liability and intellectual property. Ethics must remain at the forefront in the ever-evolving realm of healthcare technology. By embracing these strategies and best practices, healthcare systems and professionals can harness the potential of AI and robotics, ensuring responsible and ethical integration that benefits patients while upholding the highest ethical standards.

## 1. Introduction

Integrating Artificial Intelligence (AI) and robotics in healthcare is a transformative development with immense promise for revolutionizing patient care, diagnostics, and treatment modalities.^[1]^ These technologies can enhance the precision and efficiency of medical practices, improve patient outcomes, and alleviate the burden on healthcare professionals.^[2]^ However, as AI and robotics become increasingly integrated into healthcare systems, a critical set of ethical implications emerges.^[3]^ These implications touch upon privacy, data security, accountability, transparency, fairness, and the preservation of human autonomy. Understanding and addressing these ethical considerations are imperative to harness the full potential of AI and robotics in healthcare while ensuring the well-being and trust of patients, healthcare professionals, and society at large.^[4]^

This manuscript delves into the multifaceted ethical landscape surrounding AI and robotics adoption in healthcare. It critically examines the benefits and challenges of these technologies, explores the need for robust ethical frameworks, and offers insights into how healthcare systems can navigate this evolving terrain responsibly.^[5]^ By scrutinizing the ethical implications at the intersection of cutting-edge technology and patient care, this manuscript aims to contribute to developing a comprehensive ethical framework that fosters the responsible use of AI and robotics in healthcare.^[6]^

In the following pages, we will explore the ethical considerations of AI and robotics in healthcare, addressing issues such as privacy and data security, bias and fairness, accountability and transparency, autonomy and human oversight, and the societal implications of these technologies.^[7]^ We will also delve into the regulatory and legal challenges that must be overcome to ensure that AI and robotics are integrated into healthcare systems ethically and effectively.^[8]^

By examining these ethical dimensions, this manuscript seeks to provide a comprehensive understanding of the complex ethical landscape surrounding AI and robotics in healthcare.^[9]^ Ultimately, it aims to guide researchers, healthcare practitioners, policymakers, and technologists toward responsible and ethically sound practices in this transformative field.^[10]^

## 2. Objective of study

This study examines and analyzes the ethical implications of artificial intelligence (AI) and robotics in healthcare. The study aims to explore the multifaceted ethical considerations that arise from integrating AI and robotics in healthcare settings and provide a comprehensive understanding of the challenges and opportunities associated with these emerging technologies. Specifically, the study aims to:

Investigate the ethical dimensions of AI and robotics in healthcare, including privacy and data security, bias and fairness, accountability and transparency, autonomy and human oversight, impact on healthcare professionals, societal implications, and regulatory challenges.

Evaluate the potential risks and benefits of AI and robotics in healthcare ethically, considering the impact on patients, healthcare professionals, and the broader healthcare system.

Examine existing ethical frameworks, guidelines, and principles proposed to address the ethical implications of AI and robotics in healthcare and assess their adequacy and applicability in real-world healthcare settings.

Identify gaps and areas requiring further research and development in ethical practices and guidelines for the responsible integration of AI and robotics in healthcare.

Provide recommendations and insights for policymakers, healthcare organizations, and stakeholders on navigating the ethical challenges of AI and robotics in healthcare, including developing regulatory frameworks, professional training and education, and promoting transparency and accountability.

By fulfilling these objectives, this study aims to contribute to the ongoing dialogue surrounding the ethical implications of AI and robotics in healthcare and provide guidance for responsible and ethical practices in adopting and utilizing these technologies in healthcare settings.

## 3. Review

### 3.1. Benefits of AI and robotics in healthcare

The advent of AI and robotics in the healthcare sector has ushered in a new era of innovation and efficiency. These technologies offer a multitude of benefits that have the potential to significantly enhance patient care, improve healthcare outcomes, and streamline various healthcare processes. This section will explore some of the essential advantages of AI and robotics in healthcare.

#### 3.1.1. Enhanced diagnosis and treatment capabilities.

1. *Precision Medicine*: AI-driven algorithms can analyze vast datasets, including genomic information, to tailor treatment plans to individual patients. This approach, known as precision medicine, allows for more accurate diagnosis and personalized treatment strategies.^[11]^
2. *Early Disease Detection*: AI-powered diagnostic tools can detect diseases at their earliest stages by identifying subtle patterns and anomalies in medical images and patient data. This early detection can lead to better outcomes and lower treatment costs.^[12]^
3. *Clinical Decision Support*: AI systems provide healthcare professionals with real-time clinical decision support, offering evidence-based recommendations for treatment plans, drug interactions, and diagnostic accuracy. This assists clinicians in making more informed decisions.^[13]^

#### 3.1.2. Improved patient care and outcomes.

1. *Remote Monitoring*: AI and robotics enable remote patient monitoring, allowing healthcare providers to track patients’ vital signs, adherence to treatment plans, and overall health status. This reduces hospital readmissions and enhances the quality of care for chronic conditions.^[14]^
2. *Surgical Precision*: Robotic-assisted surgeries provide unparalleled precision, stability, and skill, reducing the risk of complications and accelerating patient recovery times. Surgeons can perform minimally invasive procedures with greater accuracy.^[15]^
3. *Personalized Rehabilitation*: Robotic devices are used in physical therapy and rehabilitation to provide personalized exercises and monitor progress. This ensures patients receive tailored rehabilitation programs, leading to better recovery outcomes.^[16]^

#### 3.1.3. Increased efficiency and productivity in healthcare processes.

1. *Automation of administrative tasks*: AI-driven chatbots and virtual assistants streamline administrative tasks, such as appointment scheduling and insurance verification, freeing healthcare staff to focus on patient care.^[17]^
2. *Enhanced imaging analysis*: AI algorithms can analyze medical images, such as X-rays and MRIs, at a rapid pace, reducing the time required for radiologists to interpret results. This expedites diagnosis and treatment planning.^[18]^
3. *Drug discovery and development*: AI accelerates drug discovery by analyzing vast datasets to identify potential drug candidates and predict their efficacy. This speeds up the research and development process, potentially leading to faster access to life-saving medications.^[19]^

## 4. Ethical considerations in AI and robotics

As the healthcare sector embraces the transformative power of AI and robotics, it must confront a complex web of ethical challenges that accompany these innovations. While AI and robotics offer tremendous potential to enhance patient care and streamline processes, they also introduce unique ethical considerations that demand careful examination and thoughtful resolution. This section delves into the ethical dimensions surrounding integrating AI and robotics in healthcare, focusing on critical areas of concern.

### 4.1. Privacy and data security

1. *Protection of patient data*: Using AI and robotics in healthcare generates vast amounts of sensitive patient data. Ensuring the privacy and security of this data is paramount, as any breach could have severe consequences for patient trust and data integrity.^[20]^

### 4.2. Responsible data handling and storage

1. *Data handling practices*: Healthcare institutions must adopt responsible practices for collecting, storing, and utilizing patient data. This includes robust data anonymization techniques, encryption, and secure data-sharing protocols to safeguard patient information.^[21]^

### 4.3. Bias and fairness

1. *Addressing algorithmic bias*: AI algorithms can inadvertently perpetuate biases in historical healthcare data, leading to disparities in diagnosis and treatment. It is essential to develop algorithms that mitigate bias and promote fairness in healthcare decisions.^[22]^

### 4.4. Ensuring fairness in AI-based decision-making

1. *Transparency and explainability*: Transparent decision-making processes are vital in healthcare AI. Patients and healthcare providers must understand the rationale behind AI-driven recommendations, fostering trust and accountability.^[23]^

### 4.5. Accountability and transparency

1. *Clear responsibility for AI systems’ actions*: Establishing accountability in AI and robotic systems is challenging but crucial. Determining who is responsible for errors or adverse events is essential for ethical use.^[24]^

### 4.6. Transparent decision-making processes

1. *Ethical guidelines and frameworks*: Clear ethical guidelines and frameworks should inform decision-making processes. These should be accessible to all stakeholders and regularly updated to address emerging ethical challenges.^[3]^

## 5. Ethical dimensions in greater detail: navigating the complex terrain of AI and robotics in healthcare

The ethical dimensions surrounding the integration of AI and robotics in healthcare are multifaceted and require in-depth examination to ensure responsible and ethical use. This section delves into these ethical considerations in greater detail, providing specific strategies and best practices for navigating this intricate terrain.

### 5.1. Privacy and data security

1. *Protection of patient data:*
  - *Strategy*: Implement robust encryption methods to secure patient data during transmission and storage. Adhere to internationally recognized data security standards, such as Health Insurance Portability and Accountability Act in the United States or General Data Protection Regulation in the European Union.^[25]^
  - *Best practice*: Regularly audit and update security protocols to adapt to evolving cybersecurity threats. Train healthcare staff in data security best practices to prevent breaches caused by human error.^[25,26]^

### 5.2. Responsible data handling and storage

1. Data handling practices:
  - *Strategy*: Anonymize patient data whenever possible to protect individual privacy. Consider adopting federated learning approaches that allow AI models to be trained on decentralized data without centralizing sensitive information.^[27]^
  - *Best practice*: Develop data handling guidelines specific to AI and robotics in healthcare, outlining procedures for data collection, storage, and sharing. Regularly assess data handling practices to ensure compliance with ethical standards.^[28]^

### 5.3. Bias and fairness

1. Addressing algorithmic bias:
  - *Strategy*: To mitigate bias, employ diverse and representative datasets during AI model training. Continuously monitor AI algorithms for bias and recalibrate them as necessary.^[29]^
  - *Best practice*: Collaborate with interdisciplinary teams, including ethicists and sociologists, to evaluate the potential societal impact of AI systems and identify bias in decision-making processes.^[30]^

### 5.4. Ensuring fairness in AI-based decision-making

1. Transparency and explainability:
  - *Strategy*: Develop AI algorithms with built-in transparency features, enabling healthcare providers to understand the rationale behind recommendations.^[31]^
  - *Best practice*: Implement explainable AI techniques that provide clear, understandable explanations for AI-driven decisions, enhancing trust and accountability.^[32]^

### 5.5. Accountability and transparency

1. Clear responsibility for AI systems’ actions:
  - *Strategy*: Establish transparent chains of responsibility for AI and robotic systems, delineating the roles and duties of manufacturers, healthcare institutions, and healthcare professionals.^[33]^
  - *Best practice*: Develop incident reporting mechanisms allowing healthcare professionals to report AI system errors or adverse events promptly. Create a culture of accountability within healthcare organizations.^[34]^

### 5.6. Transparent decision-making processes

1. Ethical guidelines and frameworks:
  - *Strategy*: Develop and disseminate comprehensive ethical guidelines and frameworks that inform decision-making processes involving AI and robotics in healthcare.^[35]^
  - *Best practice*: Regularly review and update these guidelines to address emerging ethical challenges. Ensure accessibility and comprehensibility for all stakeholders, including patients.^[32]^

## 6. Impact on healthcare professionals

The incorporation of AI and robotics into healthcare systems not only transforms patient care but also reshapes the roles and responsibilities of healthcare professionals. In this section, we examine the profound impact of AI and robotics on the healthcare workforce and explore the ethical considerations that arise as healthcare professionals navigate this evolving landscape.

### 6.1. Changing roles and job displacement

1. *Redefined roles*: AI and robotics redefine the roles of healthcare professionals. For instance, radiologists may transition from image interpretation to supervising AI algorithms that assist in diagnostics, requiring new skill sets and responsibilities.^[36]^
2. *Job displacement*: Automating specific tasks may raise concerns about job displacement among healthcare professionals. Ethical considerations involve ensuring a smooth transition for affected individuals and providing retraining opportunities.^[37]^

### 6.2. Collaboration between AI/robotics and healthcare professionals

1. *Interdisciplinary collaboration*: AI and robotics foster multidisciplinary collaboration between healthcare professionals and technologists. Ethical frameworks should promote effective teamwork, with clear roles and responsibilities.^[38]^
2. *Human-machine synergy*: Ethical considerations include optimizing the synergy between healthcare professionals and AI/robotic systems to leverage both strengths while preserving human expertise and oversight.^[39]^

### 6.3. Ethical considerations for healthcare professionals working with AI/robotics

1. *Professional responsibility*: Healthcare professionals are ethically responsible for understanding and appropriately using AI and robotic systems, ensuring patient safety, and remaining vigilant for system errors or biases.^[40]^
2. *Continual education*: Ethical frameworks should promote ongoing education and training for healthcare professionals to remain current with AI and robotic advancements and maintain competence.^[24]^

Integrating AI and robotics into healthcare presents opportunities and challenges for healthcare professionals. While these technologies can enhance diagnostic accuracy, streamline workflows, and improve patient care, healthcare professionals must adapt to new roles and collaborate effectively with AI and robotic systems. Ethical considerations underpin these changes, emphasizing the need for responsible deployment, education, and a focus on patient well-being.

## 7. Societal implications

Integrating AI and robotics in healthcare extends beyond clinical settings, profoundly impacting society. In this section, we delve into the societal implications of AI and robotics in healthcare, including issues related to accessibility and equity, potential exacerbation of existing inequalities, and societal acceptance and trust in these transformative technologies.

### 7.1. Accessibility and equity in healthcare

1. *Enhanced accessibility*: AI-driven telemedicine and remote monitoring offer improved healthcare access to underserved or remote populations, addressing geographic disparities in healthcare delivery.^[41]^
2. *Healthcare deserts*: Ethical concerns arise when AI and robotics inadvertently create healthcare deserts by excluding vulnerable populations who lack access to technology or digital literacy.^[42]^

### 7.2. Potential exacerbation of existing inequalities

1. *Data bias and inequalities*: AI algorithms trained on biased datasets may exacerbate healthcare inequalities, as historically disadvantaged groups may receive suboptimal care or face increased barriers to access.^[43]^
2. *Digital divide*: The digital divide, the gap in access to digital technologies, may disproportionately affect marginalized communities. Ethical considerations include strategies to bridge this divide and ensure equal access to AI-driven healthcare.^[44]^

### 7.3. Social acceptance and trust in AI and robotics

1. *Building trust*: Establishing trust in AI and robotics is essential for societal acceptance. Ethical frameworks should prioritize transparency, explainability, and user-centered design to engender public trust.^[45]^
2. *Ethical marketing*: Ethical marketing of AI and robotic healthcare solutions ensures that societal expectations align with the capabilities of these technologies, preventing unrealistic or misleading portrayals.^[46]^

The societal implications of AI and robotics in healthcare are multifaceted, with the potential to enhance accessibility and equity while also introducing new challenges related to bias and trust. Ethical considerations should guide the development and deployment of these technologies to ensure that they benefit all members of society, regardless of their socio-economic status or location.

## 8. Regulatory and legal challenges

Integrating AI and robotics in healthcare presents myriad regulatory and legal challenges requiring careful consideration. In this section, we explore the complex landscape of regulations governing AI and robotics in healthcare, liability and accountability frameworks, and issues related to intellectual property and ownership of these transformative technologies.

### 8.1. Developing robust regulations for AI and robotics in healthcare

1. *Regulatory frameworks*: The rapid evolution of AI and robotics in healthcare requires adaptable and comprehensive regulatory frameworks. Ethical considerations include the need for regulations that balance innovation with patient safety.^[47]^
2. *Interoperability standards*: Ethical regulations should emphasize the importance of interoperability standards to ensure that AI and robotic systems can seamlessly integrate with existing healthcare infrastructure.^[48]^

### 8.2. Liability and accountability frameworks

1. *Defining responsibility*: Determining liability in cases of AI or robotic errors is challenging. Ethical considerations include the establishment of clear accountability frameworks that allocate responsibility among manufacturers, healthcare institutions, and healthcare professionals.^[49]^
2. *Informed consent*: Ethical frameworks should address the issue of informed consent, mainly when AI or robotic systems are involved in decision-making. Patients must be informed about the roles of these technologies in their care.^[50]^

### 8.3. Intellectual property and ownership of AI/robotic technologies

1. *Patents and innovation*: Ethical considerations in intellectual property emphasize the balance between incentivizing innovation through patents and ensuring that AI and robotic technologies remain accessible for widespread healthcare benefits.^[51]^
2. *Ownership and data rights*: Defining ownership and rights in AI-generated medical data is complex. Ethical frameworks should prioritize equitable data sharing and rights while respecting privacy.^[52]^

Navigating the regulatory and legal challenges surrounding AI and robotics in healthcare is essential for responsible and ethical integration. Balancing innovation with safety, defining liability, and addressing intellectual property and data ownership issues are key ethical imperatives in ensuring that AI and robotics contribute positively to healthcare.

## 9. Ethical frameworks and guidelines

In the dynamic landscape of AI and robotics in healthcare, the development and application of ethical frameworks and guidelines are crucial to ensure responsible and ethical use. In this section, we delve into the existing ethical frameworks that provide essential guidance for the ethical integration of AI and robotics in healthcare and emphasize the need for comprehensive and adaptable guidelines tailored to address the unique challenges posed by these technologies.

### 9.1. Existing ethical frameworks for AI and robotics in healthcare

1. *Principles of medical ethics*: Medical ethics, such as autonomy, beneficence, non-maleficence, and justice, remain foundational in guiding ethical practices in AI and robotics in healthcare. These principles underscore the importance of patient welfare and equitable care.^[1,53,54]^
2. *The Belmont report*: The Belmont Report’s principles of respect for persons, beneficence, and justice are highly relevant in AI and robotics. They emphasize the importance of informed consent, the promotion of well-being, and the fair distribution of benefits and burdens.^[2]^
3. *IEEE ethically aligned design*: The IEEE’s Ethically Aligned Design framework provides comprehensive guidance for the ethical development and deployment of autonomous and intelligent systems, including AI and robotics. It emphasizes transparency, accountability, and the prioritization of human values.^[3]^

### 9.2. Need for comprehensive and adaptable guidelines

1. *Healthcare-specific guidelines*: While existing frameworks offer valuable guidance, healthcare-specific guidelines are needed to address the unique ethical considerations in this domain. These guidelines should consider patient-doctor relationships, diagnostic accuracy, and data privacy.^[4]^
2. *Adaptability*: Ethical guidelines must be adaptable to the rapidly evolving field of AI and robotics. They should incorporate mechanisms for continuous evaluation and updates to address emerging ethical challenges.^[55]^
3. *Global collaboration*: Collaboration among international stakeholders is essential to develop guidelines that reflect diverse cultural, legal, and ethical perspectives. Global consensus can foster responsible and ethical use worldwide.^[6,56]^

Developing and adhering to ethical frameworks and guidelines are instrumental in guiding the responsible integration of AI and robotics into healthcare. These frameworks draw from established ethical principles and are essential for ensuring that these technologies are used to benefit patients while upholding ethical standards.

## 10. Conclusion

Integrating AI and robotics into healthcare represents a monumental shift in the field, promising improved diagnostics, treatments, and healthcare delivery. However, this transformative journey is accompanied by a complex landscape of ethical considerations that demand careful navigation. In this concluding section, we summarize the key insights from this manuscript and underscore the importance of a balanced and ethical approach in harnessing the potential of AI and robotics in healthcare.

The ethical implications of AI and robotics in healthcare touch upon various critical dimensions:

1. *Privacy and data security*: Protecting patient data is paramount, necessitating robust data handling and storage practices to ensure privacy and security.
2. *Bias and fairness*: Addressing algorithmic bias is essential to prevent disparities in diagnosis and treatment, promoting fairness in healthcare decision-making.
3. *Accountability and transparency*: Establishing clear responsibility for AI systems’ actions and transparent decision-making processes are crucial for trust and ethical use.
4. *Autonomy and human oversight*: Maintaining human control over AI and robotic systems while preserving patient autonomy and consent is an ethical imperative.
5. *Impact on healthcare professionals*: Changing roles and job displacement require ethical considerations, as does fostering effective collaboration between AI/robotics and healthcare professionals.
6. *Societal implications*: Ethical concerns related to accessibility, equity, and societal trust must guide the responsible deployment of these technologies.
7. *Regulatory and legal challenges*: Developing adaptable regulations and liability frameworks and addressing intellectual property issues are essential for ethical integration.
8. *Ethical frameworks and guidelines*: Existing ethical frameworks provide guidance, but healthcare-specific, adaptable guidelines are needed to navigate this evolving landscape.

In conclusion, AI and robotics in healthcare have the potential to revolutionize patient care and healthcare delivery. However, their responsible and ethical use is contingent on addressing the multifaceted ethical considerations outlined in this manuscript. By prioritizing patient welfare, transparency, fairness, and collaboration, healthcare systems, technologists, policymakers, and healthcare professionals can ensure that AI and robotics contribute positively to healthcare while upholding the highest ethical standards.

As we move forward, we must recognize that the ethical journey in AI and robotics in healthcare is ongoing. Continuous reflection, adaptation, and collaboration will be essential to harness the full potential of these technologies while safeguarding patient well-being and trust.

In the ever-evolving healthcare technology landscape, ethics must remain at the forefront to ensure that AI and robotics are tools for healing and empowerment, benefiting individuals and society.

## Author contributions

**Conceptualization:** Chukwuka Elendu, Dependable C. Amaechi, Tochi C. Elendu, Klein A. Jingwa, Minichimso John Okah, John A. Ladele.

**Data curation:** Chukwuka Elendu, Dependable C. Amaechi, Tochi C. Elendu, Osinachi K. Okoye.

**Formal analysis:** Chukwuka Elendu, Dependable C. Amaechi, Tochi C. Elendu, John A. Ladele.

**Funding acquisition:** Dependable C. Amaechi, Tochi C. Elendu.

**Investigation:** Chukwuka Elendu, Dependable C. Amaechi, Tochi C. Elendu, Klein A. Jingwa, Abdirahman H. Farah.

**Methodology:** Chukwuka Elendu, Dependable C. Amaechi, Tochi C. Elendu.

**Project administration:** Chukwuka Elendu, Dependable C. Amaechi, Tochi C. Elendu, Minichimso John Okah.

**Resources:** Chukwuka Elendu, Dependable C. Amaechi, Tochi C. Elendu, Abdirahman H. Farah.

**Software:** Chukwuka Elendu, Dependable C. Amaechi, Tochi C. Elendu, Klein A. Jingwa.

**Supervision:** Chukwuka Elendu, Dependable C. Amaechi, Tochi C. Elendu, Klein A. Jingwa, John A. Ladele, Hameed A. Alimi.

**Validation:** Chukwuka Elendu, Dependable C. Amaechi, Tochi C. Elendu, Klein A. Jingwa, Osinachi K. Okoye, Minichimso John Okah, Hameed A. Alimi.

**Visualization:** Chukwuka Elendu, Dependable C. Amaechi, Tochi C. Elendu, Osinachi K. Okoye, Minichimso John Okah.

**Writing – original draft:** Chukwuka Elendu, Dependable C. Amaechi, Tochi C. Elendu, Minichimso John Okah.

**Writing – review & editing:** Chukwuka Elendu, Dependable C. Amaechi, Tochi C. Elendu, Osinachi K. Okoye, Minichimso John Okah, Abdirahman H. Farah, Hameed A. Alimi.
